# Retronasal odor concentration coding in glomeruli of the rat olfactory bulb

**DOI:** 10.3389/fnint.2014.00081

**Published:** 2014-10-24

**Authors:** Shree Hari Gautam, Shaina M. Short, Justus V. Verhagen

**Affiliations:** ^1^The John B. Pierce LaboratoryNew Haven, CT, USA; ^2^Department of Neurobiology, Yale University School of MedicineNew Haven, CT, USA

**Keywords:** retronasal odor, olfactory bulb, optical imaging, odor concentration, glomerular dynamics, concentration response function

## Abstract

The mammalian olfactory system processes odorants presented orthonasally (inhalation through the nose) and also retronasally (exhalation), enabling identification of both external as well as internal objects during food consumption. There are distinct differences between ortho- and retronasal air flow patterns, psychophysics, multimodal integration, and glomerular responses. Recent work indicates that rats can also detect odors retronasally, that rats can associate retronasal odors with tastes, and that their olfactory bulbs (OBs) can respond to retronasal odorants but differently than to orthonasal odors. To further characterize retronasal OB input activity patterns, experiments here focus on determining the effects of odor concentration on glomerular activity by monitoring calcium activity in the dorsal OB of rats using a dextran-conjugated calcium-sensitive dye *in vivo*. Results showed reliable concentration-response curves that differed between odorants, and recruitment of additional glomeruli, as odor concentration increased. We found evidence of different concentration-response functions between glomeruli, that in turn depended on odor. Further, the relation between dynamics and concentration differed remarkably among retronasal odorants. These dynamics are suggested to reduce the odor map ambiguity based on response amplitude. Elucidating the coding of retronasal odor intensity is fundamental to the understanding of feeding behavior and the neural basis of flavor. These data further establish and refine the rodent model of flavor neuroscience.

## INTRODUCTION

Retronasal smell pertains to volatile stimuli released from food in the mouth while eating. These odors travel to the nasal cavity during exhalation via the nasopharynx. Orthonasal smell pertains to odors entering through the nares from the external environment. In human’s retronasal and orthonasal odorants comprise two distinct functions of olfaction (Rozin, 1982; Bender et al., 2009). These two modes of olfaction are associated with two overlapping but separate neural networks (Small et al., 2005). Studies further indicate that retronasal smell, at both threshold and suprathreshold odor concentrations, is less sensitive than orthonasal smell in humans (Heilmann and Hummel, 2004; Hummel et al., 2006; Furudono et al., 2013). These sensitivity differences may in part be explained by difference in direction-dependent flow patterns across the olfactory epithelium (Zhao et al., 2006) in interaction with flow rate and non-uniform receptor distributions (Schoenfeld and Cleland, 2006), in addition to differences in higher level mechanisms as learning (Bender et al., 2009).

The human olfactory system is well adapted to encoding not only odor identities, but also odor intensities of the volatiles present in food (Small et al., 2005). In humans, odor concentration has been shown to be directly proportional to perceived odor intensity, as well as olfactory receptor neuron population responses (Lapid et al., 2009). While odor identity coding during orthonasal smell has been fairly well studied (Stewart et al., 1979; Rubin and Katz, 1999; Meister and Bonhoeffer, 2001; Spors et al., 2006; Verhagen et al., 2007), and ignored odor direction, studies on the retronasal odor identity coding have only recently begun (Scott et al., 2007; Gautam and Verhagen, 2012b; Furudono et al., 2013). Furthermore, a systematic study of the coding of retronasal odor intensities in the olfactory bulb (OB) of any species is yet to be reported. The purpose of this study was to map retronasal odor intensity coding at the inputs of the first synaptic relay, the glomeruli, in the dorsal OB of the rat, a model of flavor neuroscience.

Spatiotemporal activity patterns of glomeruli in the OB is believed to code information pertaining to odor identity (Spors and Grinvald, 2002; Spors et al., 2006). The activity of each glomerulus corresponds to stimulation of specific olfactory receptor neurons. During odor presentation a unique glomerular activation pattern is created, often referred to as an odor map, which presumably contains information pertaining to all activated olfactory receptor neurons. It has been frequently reported that, upon increasing the concentration of an orthonasal odor both glomerular response magnitude and number of glomeruli recruited significantly increase (Rubin and Katz, 1999; Johnson and Leon, 2000; Meister and Bonhoeffer, 2001; Wachowiak and Cohen, 2001; Spors et al., 2006; Homma et al., 2009; Vincis et al., 2012). However, the features of OB glomerular activity patterns that code for odor intensity have not been studied and reported systematically to date for retronasal smell, except for 1 odor in 1 mouse (see Figure 3 in Furudono et al., 2013).

Across species, behavioral assessments indicate that the olfactory system is capable of recognizing odors when presented at a large range of concentrations (Slotnick and Ptak, 1977; Stopfer et al., 2003). Additionally, orthonasally stimulated OB activity maps depend on odor concentration (Johnson and Leon, 2000; Wachowiak et al., 2002; Stopfer et al., 2003; Lecoq et al., 2009). Increases in both detection accuracy and OB response magnitudes are observed with increases in odor concentration. It remains to be tested if this holds for retronasal odors as well.

Experiments presented here focus on understanding how glomerular responses to retronasal stimulation code for odor concentration by monitoring calcium activity presynaptically in the anesthetized rat. Previous work from our lab has shown that the rat also can perceive retronasal odorants (Gautam and Verhagen, 2012a) and form odor-taste flavors like humans (Gautam and Verhagen, 2010). We further showed that retronasal OB response magnitudes are about half of their orthonasal counterparts, the ratio of which depends on an odor’s specific vapor pressure (Gautam and Verhagen, 2012b). Here we systematically studied the interaction between four concentration levels of up to five odorants with up to six repetitions per rat for high statistical power (**Tables 1A,B**; which extensive protocol precluded us from testing orthonasal stimulation). We also performed new across-glomerular pattern comparisons across odor routes based on our previously published data (Gautam and Verhagen, 2012b). Our results show unequivocally that retronasal odor concentration directly influences the magnitude of glomerular responses as well as total number of recruited glomeruli. This work sheds light on the complexity with which odor qualities and concentrations are processed retronasally in the OB by providing the first systematic quantitative assessment of retronasal odor concentration across odorants on glomerular activity levels and dynamics.

**Table 1:** The effect of concentration depends on the odor.

**(A) Data trials summary**
	rat 1 (rac110)	rat 2 (rac111)	rat 3 (rac112)	rat 4 (rac121)	rat 5 (rac124)	rat 6 (rac126)	rat 7 (rac127)	rat 8 (rac129)	rat 9 (rac130)

2-butanone (2-But)	4 X (2-3)	**–**	–	–	–	4 X (3-4)	4 X (2-4)	4 X(4-5)	**–**
Hexanal (Hexa)	4 X (2-3)	**–**	3 X(1-5)	4X (4)	4 X (2-4)	4 X (3-6)	**–**	4X(4)	4 X (4-6)
Ethyl butyrate (EB)	4 X (3-5)	4 X (3-5)	4 X (3-6)	4 X (2-7)	**–**	4 X (3-6)	4 X (4-6)	4X(4)	4 X (4-6)
Methyl valerate (MV)	4 X (3-4)	**–**	4 X (3-4)	**–**	**–**	**–**	4 X (2-4)	4 X (4-6)	4 X (4)
Amyl acetate (AA)	4 X (3)	**–**	4 X (3)	**–**	**–**	4 X (3-4)	**–**	**–**	4 X (4-6)

**(B) MANOVA P values**
	**rat 1(22)**	**rat 2(16)**	**rat 3(29)**	**rat 4(18)**	**rat 5(17)**	**rat 6(3)**	**rat 7(8)**	**rat 8(23)**	**rat 9(29)**	%

Odor	***	***	*	***	***	**	***	***	***	100
Cone	***	***	*	*	**	***	***	***	***	100
Odor × concentration	***	***	*	*	***	*	***	***	***	100

	*** =<0.001		** = <0.01		* = <0.05		

***(A)** A summary of the data collected. The odorants tested and the number of odor stimulation trials at different odor concentrations (0.3, 1, 3, 10) per experimental rat (number of concentrations X range of repetitions; rat lab code in parentheses; boldfaced rats used for across-glomerular pattern analyses). **(B)** MANOVA of effects of odor and concentration on response magnitude for each animal (number of significant glomeruli in parentheses).*

## MATERIALS AND METHODS

### LABELING OLFACTORY RECEPTOR NEURONS

Methods used in this study closely follow previous work (Gautam and Verhagen, 2012b). Initially, olfactory receptor neurons in the dorsal recess of the nasal cavity of Long-Evans female rats were loaded bilaterally with dextran-conjugated calcium-sensitive dye (Oregon Green BAPTA 488-1 dextran; Invitrogen, Carlsbad, CA, USA) using a well-established protocol (Wachowiak and Cohen, 2001), adapted for rats (Verhagen et al., 2007). Nine animals were held 8–17 days before recording. These rats weighed 180–200 *g* and were purchased from Charles River Laboratories Inc. (New York, NY, USA) and housed individually. All the animals were treated according to the guidelines established by the U.S. National Institutes of Health (1986). The experimental protocols were approved by the Institutional Animal Care and Use Committee of the John B. Pierce Laboratory. The John B. Pierce Laboratory is AAALAC accredited.

### OPTICAL WINDOW AND DOUBLE TRACHEOTOMY SURGERY

**FIGURE 1:**
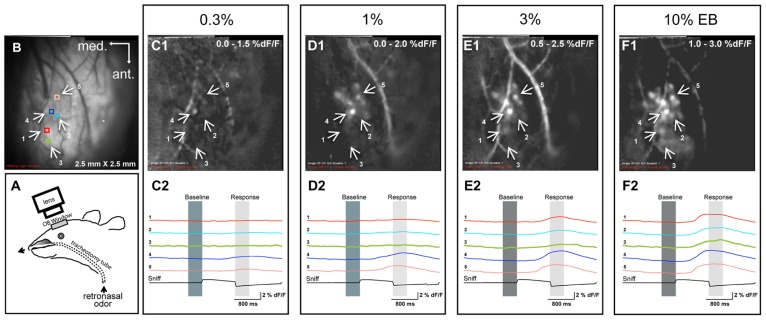
**Examples of retronasal odor responses to different odor concentrations. (A)** Experimental setup for retronasal delivery of odorants during optical imaging of the olfactory bulb. The olfactometer infuses odorants directly into the nasopharynx. **(B)** Resting light intensity (RLI) of the left bulb and positions of five regions of interest (ROIs), also shown in **C1–F1**, which display sniff-triggered average odor maps (% dF/F; *n* = 3 presentations for each map) evoked by different concentrations of ethyl butyrate (EB). **(C2–F2)** Responses displayed across time at five different ROIs for each concentration.

Prior to imaging, the dye-infused rats were anesthetized with urethane (1.5 g/kg i.p.), the bone overlying the dorsal surface of the bulb was exposed, thinned and coated with cyanoacrylate glue to make the bone transparent (Bozza et al., 2004). A double tracheotomy surgery was performed as described previously (Gautam and Verhagen, 2012b) allowing for the rat to sniff artificially and to breathe through the trachea. Briefly, a Teflon tube (OD 2.1 mm, upper tracheotomy tube) was inserted 10 mm into the nasopharynx, to assure that airflow was restricted to the nose (the epiglottis could otherwise leak air flow via the oral cavity). Another Teflon tube (OD 2.3 mm, lower tracheotomy tube) was inserted in to the caudal end of the tracheal cut. Both tubes were fixed and sealed to the tissues using surgical thread and cyanoacrylate glue. The head was stabilized by gluing it to a bar mounted on a stereotaxic head holder designed not to interfere with tracheal breathing. The upper tracheotomy tube inserted into the nasopharynx was used to deliver odor stimuli retronasally (**Figure 1A**). Local anesthetic (2% Lidocaine) was applied at all pressure points and incisions. Artificial sniffing was synchronized to the start of each trial. Throughout the surgery and optical recordings rats’ core body temperature was maintained at 37°C with a thermostatically controlled heating pad (Omega Engineering Inc., Stamford, CT, USA).

### OPTICAL RECORDINGS

Optical calcium signals from the dorsal OB were recorded using a CCD camera (Redshirt Imaging LLC, Decatur, GA, USA) with 256 × 256 pixel resolution, and at a frame rate of 25 Hz. This resolution was sufficient to identify single glomeruli at magnifications low enough to image across the dorsal surface of the bulb. The epifluorescence macroscope used was a custom-made tandem-lens type (Ratzlaff and Grinvald, 1991) with 2× magnification and high NA (0.85–0.95) CCTV objectives for high SNR. A high power LED (Luxeon LXHL-PE09, Philips Lumileds, San Jose, CA, USA) driven by a linear DC power supply acted as the light source. A custom-made DC amplifier (based on a linear Apex power operational amplifier; Cirrus Logic, Inc., Austin, TX, USA) powered a peltier (Melcor, OT2.0-31-F1) device onto which the LED was glued. The LED-cooling peltier current was proportional to the LED current, yielding a stable illumination. The fluorescence filter set used was BL P01-514 (excitation filter), LP515 (dichroic), and LP530 (emission filter; Semrock, Lake Forest, IL, USA). This system provided fast imaging capabilities, a large field of view, and low noise. Raw images were converted to images representing the relative change in fluorescence (%Δ*F*/*F*) in each pixel and frame after stimulus application. Data analysis was performed using NeuroPlex software (RedShirtImaging LLC, Decatur, GA, USA), and routines were written in Matlab (Version 7.11.0, The MathWorks Inc., Natick, MA, USA).

### RETRONASAL STIMULATION AND ODORANTS

The schematics of the experimental setup and examples of retronasal imaging trials are shown in **Figure 1**. We modified our previously used bi-directional artificial sniffing paradigm (Gautam and Verhagen, 2012b) to an unidirectional retronasal artificial paradigm by removing the nose mask (**Figure 1A**). The olfactometer infused odorants directly into the nasopharynx, which was made accessible via the double tracheotomy. This retronasal artificial sniffing paradigm by positive-pressure was also connected to a pressure sensor (Honeywell, Morristown, NJ, USA; part 24PCAFA6G) to measure the flow-resistance, which enabled us to properly control the delivery of the odor stimuli retronasally. The time to fill the dead volume was ∼120 ms. We chose a flow rate of 250 ml/min as this was found by Youngentob et al. (1987) to be the average flow rate of in- and expiratory sniffing by awake behaving rats (see their **Table 2**, 1.9–8.9 ml/s). The Teflon valves (NResearch Inc., West Caldwell, NJ, USA) involved in this paradigm were automated by a program written in Labview (National Instruments, Austin, TX, USA). All the results are based on the responses to the first odor pulse only unless otherwise stated.

**Table 2:** The effect of odor concentration on temporal response parameters.

Odorant	P	VP	MW	Onset	t-10	t-50	t-90	t-peak
AA	2.26	5.6	130.2	**	ns	ns	ns	ns
EB	1.85	12.8	116.2	ns	**	*	ns	ns
MV	1.85	19.1	116.2	*	**	**	*	ns
hexa	1.8	11.3	100.2	ns	ns	ns	ns	ns
2but	0.26	90.6	72.11	ns	**	**	*	ns

	** = <0.01			* = <0.05					

*P -values comparing the time course of response magnitudes across odorants of all concentrations (39 odors and odor concentrations tested, 4–8 rats per odor). See **Figure 4** for temporal profiles.*

Each imaging session consisted of ∼60–130 manually triggered trials with an inter-trial interval of >3 min. The odorants were presented semi-randomly such that lower concentrations of odor were presented before higher concentrations. In each trial the same concentration of an odor was presented at 250 ml/min flow rate at each of the three 1 s pulses separated by 2.5 s interval, using a custom-built multichannel auto-switching flow dilution olfactometer (Lam et al., 2000) with dedicated lines for each odor to avoid cross-contamination. Five odorants (EB, ethyl butyrate; MV, methyl valerate; Hexa, hexanal; 2-BUT, 2-butanone; and AA, amyl acetate) were presented retronasally. This allowed for the continuous control of odor concentration over 1.5 log units. After each stimulus the nasal cavity was flushed with clean humidified (sparging distilled water) air for 1 min. The olfactometer output was routed to a set of route-switching valves that were mounted on the side of the stereotax so as to minimize the dead space. Odor concentrations are indicated as percentage saturated vapor (% s.v.). Medical-grade air was used to dilute the vapor in the headspace of odor reservoirs to generate the desired concentration. We tested up to five odors at four concentrations across typically 3–5 trials in the nine rats (**Table 1A**).

Monomolecular odorants were chosen from the family of odorants whose effects on the dorsal bulb have been previously characterized (Johnson and Leon, 2000; Uchida et al., 2000; Meister and Bonhoeffer, 2001; Wachowiak and Cohen, 2001) and predominantly activate the dorsal OB (based on our Matlab analysis of the database kindly provided by Drs. Johnson and Leon). The entire odor delivery system was made of Teflon. All the odorants were obtained from Sigma-Aldrich (St. Louis, MO, USA) and stored under nitrogen in the dark.

### MEASUREMENT OF BREATHING

Breathing was measured as the movement of the thorax by a piezoelectric strap around the animal’s chest as described previously (Gautam and Verhagen, 2012b). During each respiration cycle, one sharp upward deflection in the piezoelectric signal occurred during thorax expansion (inspiration). The point of onset of this deflection occurring before and after the stimulus onset time was used as a reference for estimating instantaneous breathing frequency and assessing occurrence of response coupling with breathing cycle. The temporal parameters were measured in reference to the stimulus onset time recorded directly by a pressure sensor connected to the artificial sniffing setup.

### DATA ANALYSIS

#### Identification of activated glomeruli

Datasets consisting of optical images of 256 × 256 pixels sampled at 25 Hz, pressure signals sampled at 200 Hz, breathing signals, information on odor identity, odor concentration and flow rate were acquired using Neuroplex software on a 12-s trial-by-trial basis. Script files written in MATLAB were used to extract data and to correct global noise in every imaged frame. The images were then averaged across trials for each stimulus to identify regions of interest (ROIs, activated glomeruli). Focal changes in fluorescence in the OB have been shown previously to correspond to individual glomeruli (Belluscio and Katz, 2001; Meister and Bonhoeffer, 2001; Bozza et al., 2004).

#### Estimation of response magnitude

Using the identified ROI we then extracted average glomerular response curves (*F*-traces) based on the stimulus onset times of five different odorants. These *F*-traces guided the selection of optimum pre-frame (before stimulus onset) and post-frame (response maximum) windows, which consisted of 15–21 frames (600–840 ms). The response magnitudes across each ROI were then measured using this window for each trial as the percent change in fluorescence before and after stimulus onset (% ΔF/F) as reported previously (Verhagen et al., 2007; Gautam and Verhagen, 2012b). Multivariate analysis of variance (MANOVA; odor × concentration) was then performed across all trials from an animal, and any ROI for which effect of odor (including odorless air, delivered via a separate clean line, which allows for testing of odor main effect in case a single odor was tested) was not significant was removed from further analyses (**Table 1B**).

#### Spatial and temporal analyses of retronasal response patterns: population-average

Peak response amplitudes (% ΔF/F) at the ROIs were compared among different concentrations of odorants. Both peak glomerular responses as well as the slopes in glomerular responses across varying odor concentrations (odor-concentration slopes) were examined. Correlation analysis and analysis of variance (ANOVA) were used to establish the effect of odor concentration on the spatial odor map. Averages are reported ± SEM (SD/√n). Alpha-level was set at 0.05.

To measure temporal parameters of the glomerular response a custom algorithm was developed that fitted the optical signals from each ROI to a double sigmoid function as described previously (Carey et al., 2009; Gautam and Verhagen, 2012b). The analysis allowed robust and objective measurement of response timing. Briefly, the signal from each ROI was band-pass filtered (second-order Butterworth, 0.1–1 Hz) followed by fourth-order Daubechies wavelet decomposition, soft thresholding of the coefficients at level 3, and then reconstruction. The onset time was defined based on the time of peak in the product of the first and the second derivatives of the optical signal. Starting at this time, each response was fitted (least-squares curve fitting) with a double-sigmoid function (a sigmoid rise followed by a sigmoid fall). The time of the peak of this response was defined as the peak in this fitted response function, rather than the peak of the raw optical signal.

**FIGURE 2:**
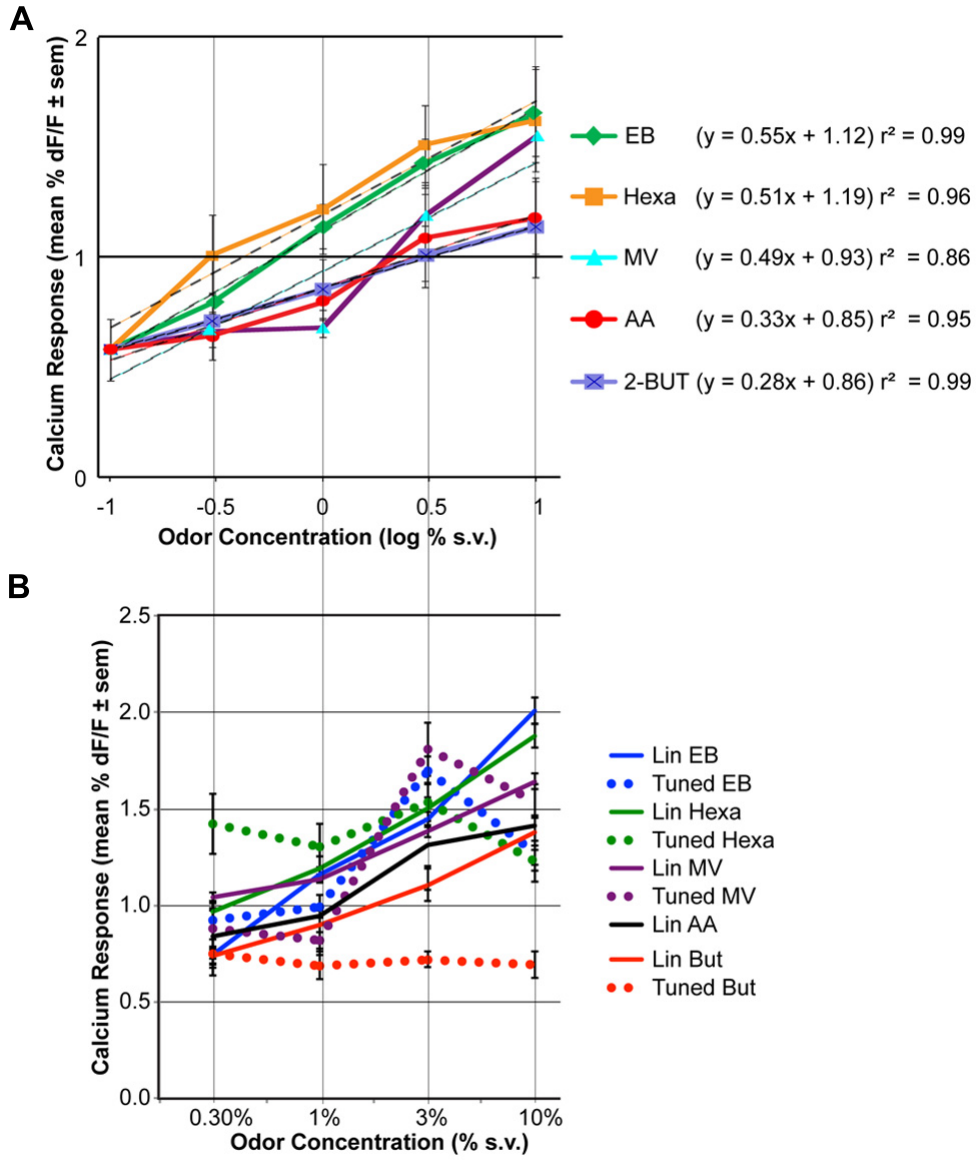
**The effect of odor concentration on glomerular response. (A)** Average concentration-response curves for five odorants. For each odor the effect of concentration was statistically significant (ANOVA, *P* < 0.05, *n* = 9). Responses to clean air are displayed at –1 log % (as this is around response measurement threshold). The equation and *r*^2^ of log-linear fits are indicated on the right. **(B)** Mean concentration-response curves of the linearly increasing (solid lines) versus tuned glomeruli (dashed lines). Glomeruli were grouped based on cluster analysis of the concentration-response pattern correlations (see Figures S1–S5 for detailed data).

**FIGURE 3:**
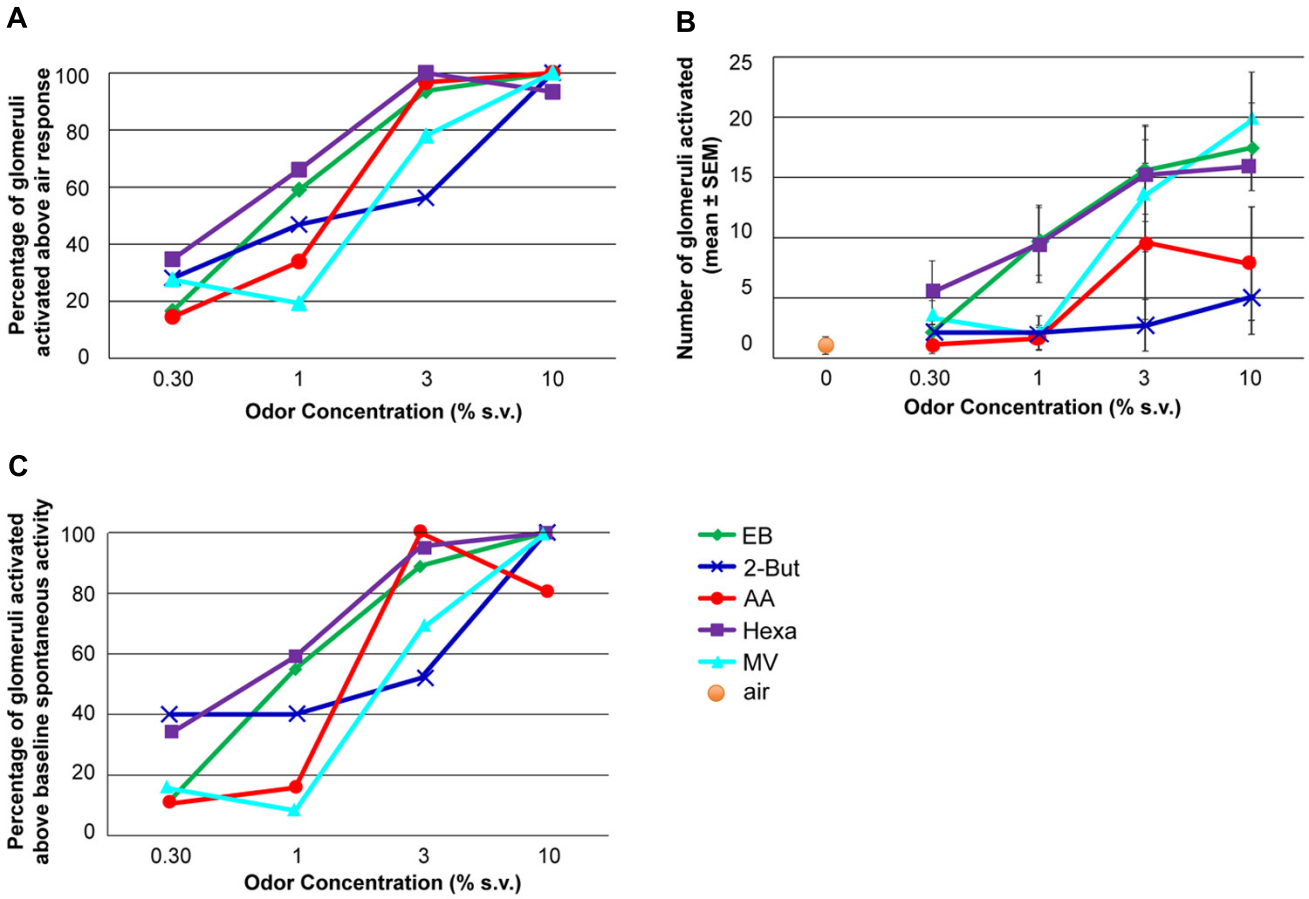
**The effect of odor concentration on glomerular recruitment. (A)** Percentage of glomeruli activated at each concentration. The maximum number of glomeruli activated by each odor constituted 100%. The activation threshold was set at the response to odor-free clean air + 1.65 SD. **(B)** Number of glomeruli activated at each concentration when the recruitment threshold was set at the response to clean air + 1.65SD. **(C)** Percent of glomeruli activated by each odor at each concentration above un-stimulated activity levels. For each graph the effect of odor concentration was significant (ANOVA, *P* < 0.05, *n* = 9 rats).

**FIGURE 4:**
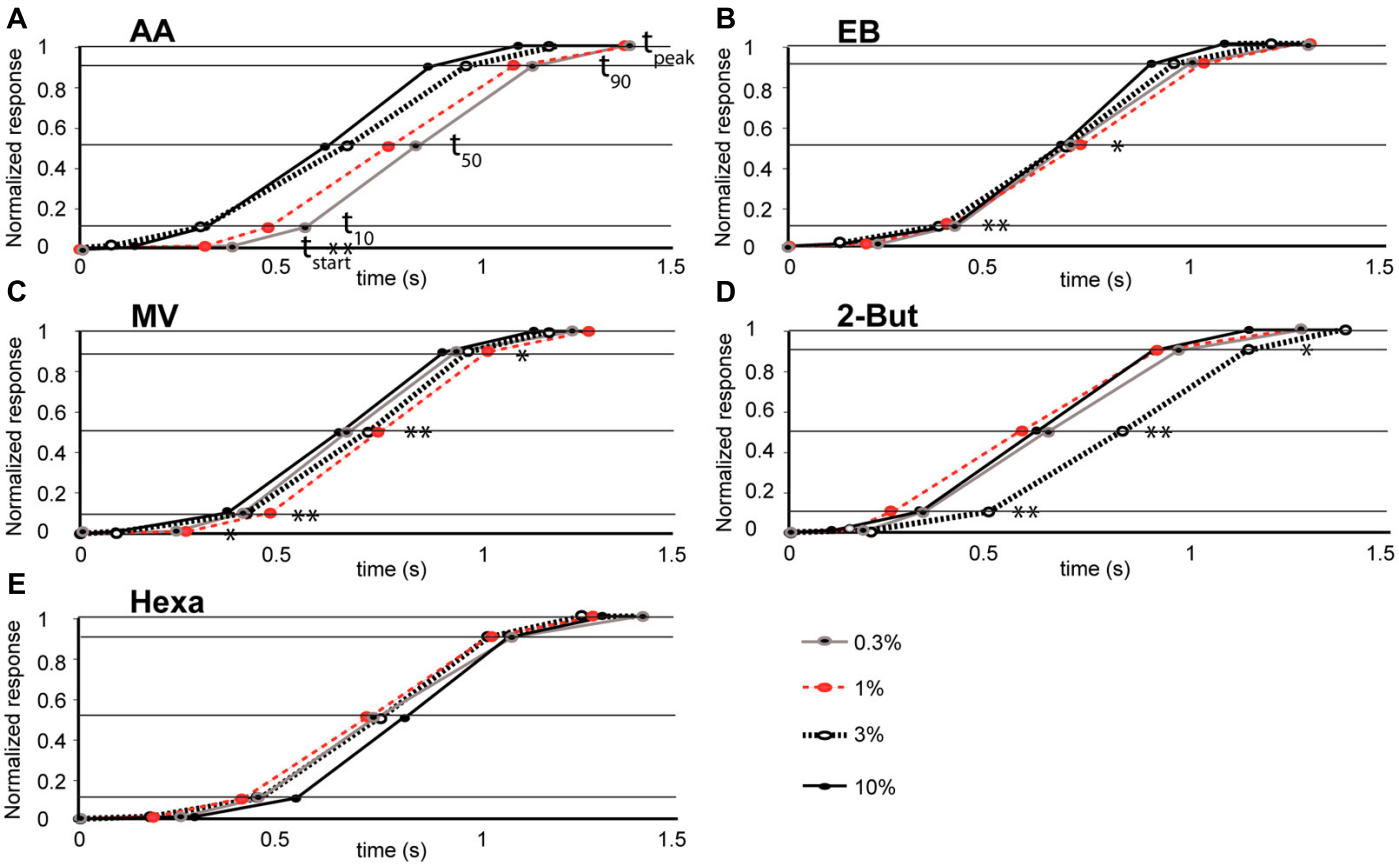
**Relationship between odor concentration and the mean time-course of retronasal odor responses **(A–E)**.** Stimulus onset time = 0 and other data points correspond to time constants (t_start_, t_10_, t_50_, t_90_, t_peak_ marked by horizontal lines) for odorants, AA (*n* = 4 rats), EB (*n* = 8), MV (*n* = 5), Hexa (*n* = 7), and 2-But (*n* = 4) respectively. Significant effects of odor concentration on the dynamic parameters are marked. *P < 0.01, **P < 0.05, see **Table 2** for ANOVA statistics.

For population analyses (**Figures 2A**, **3**, and **4**) for each stimulus at each concentration we first averaged each response parameter (magnitude and time) for each glomerulus across al trials. We next averaged these across the glomerular population for each animal.

Glomerular recruitment (**Figure 3**) was assessed as mean glomerular response above two different baseline measurements. One was based on non-stimulated signal magnitudes (“noise,” *n* = 5 rats, a subset of the nine rats for which we had non-stimulated data) and the other on responses that were evoked by clean air presentations (*n* = 8 rats). In both cases an activation was defined as the average response being above the baseline average + 1.65 SD (i.e., *P* < 0.05, see e.g., Verhagen et al., 2003).

To compare the population-averaged temporal dynamics of glomerular responses across concentrations of an odor (**Figure 4**) we extracted parameters of the time course of responses at each glomerulus. The parameters were first averaged across glomeruli for each odor and concentration separately. Then the mean for each concentration per odor was averaged across eight rats. Correlation analysis and ANOVA were used to establish whether the change in concentration affected the temporal glomerular response dynamics (**Table 2**).

#### Spatial analysis of retronasal response patterns: glomerular concentration-response profiles

We evaluated the concentration-response profiles of all glomeruli. We submitted the trial-averaged response magnitudes of all glomeruli to cluster analysis, for each odor separately (linkage: average, distance: Pearson; “matrix”; Systat 10.2, Systat Software, Inc., San Jose, CA, USA). Figures S1–S5 show the results, including response heat maps across increasing retronasal odor concentrations. By visual inspection of the heat maps we identified clusters that did (“linear”) and did not (“tuned”) show increasing concentration-response functions, the former peaking at 10% v.p., the latter below 10% v.p. Figure S6 shows all responses across all odors and concentrations, clustered by Euclidian distance (single linkage) across both glomeruli and stimuli. **Figure 2B** shows the response profiles for the clusters, averaged across the glomerular members.

#### Spatial and temporal analyses of retronasal response patterns: across glomeruli

**FIGURE 5:**
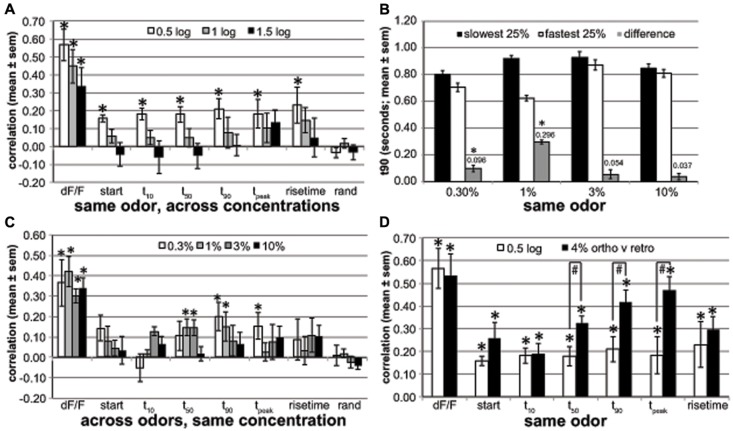
**Across-glomerular pattern similarities. (A)** The pattern similarity evoked by different concentrations (0.5, 1, and 1.5 log-units difference) of the same retronasal odor for response magnitude (dF/F) and dynamic parameters (start, t_10_, t_50_, t_90_, t_peak_ and rise time) averaged across four rats. rand: pattern similarities based on identically processed random control data. Response magnitude patterns are well preserved across concentrations, but temporal patterns are not when the concentration difference is 10× or more (*one-sided paired *t*-test for mean > 0, *P* < 0.00065, *n* = 16–50 correlations based on 103 glomeruli of four rats boldfaced in **Table 1A**). **(B)** t_90_ of glomeruli with slowest t90 versus fastest t90 quartile at 1% v.p. and their t_90_ difference. These dynamics (296 ms difference) are only mildly preserved at 0.3% v.p. with a 96 ms difference (*one-sided paired *t*-test for mean difference > 0, *P* < 0.001, *n* = 16–17 mean t_90_ values of 4–5 odors of same four rats) and not at 3 or 10% v.p. **(C)** The response magnitude and dynamic pattern similarity evoked by same concentrations (0.3–10%) across different retronasal odors averaged across four rats. Dynamics for one retronasal odor are poor predictors of those for another retronasal odor (*one-sided paired *t*-test for mean > 0, *P* < 0.00065, *n* = 25–28 correlations of 4–5 odors of same 103 glomeruli of four rats as in **A**). **(D)** Pattern similarity for 0.5-log spaced concentrations (same as in **A**) compared to pattern similarities across orthonasally and retronasally presented odorants at 4% v.p. based on a new analysis of a previously published dataset (Gautam and Verhagen, 2012b; *n* = 9 rats; *one-sided paired *t*-test for mean > 0, *P* < 10^-4^, *n* = 70 correlations from 150 glomeruli from 9 rats, 4–8 odors and air per rat; #one-sided unpaired *t*-test for 4% ortho-retro > 0.5 log, *P* < 0.02, *n* = 4 and nine rats, response; all others ns). Late dynamic patterns are fairly similar between the two odor routes, and more so than across retronasal odors that differ by 0.5 log % v.p. concentration.

We also investigated the similarities of response dynamics and magnitude across-glomerular populations (**Figure 5**). Here the derived parameters were averaged across trials for each odor at each concentration but not across glomeruli. Correlations were then established (Microsoft Excel 2010) across the glomerular population for each rat or the comparison of interest.

We calculated the correlations across glomeruli of the parameters (dF/F, t_10_, etc) between 0.5 and 1.5 log unit spaced concentrations of the same odor (**Figure 5A**). We then averaged across odors per concentration spacing. We used *n* = 16–50 correlations based on 103 glomeruli of four rats boldfaced in **Table 1A**. The number of available comparisons decreased as the spacing increased: *n* = 50 (0.5 log-unit), 33 (1 log-unit), 15 (1.5 log-unit) correlations, respectively. The *t*-tests were based on these correlations. The figure shows the mean ± SEM across four rats. For **Figure 5C** the same general approach was used, but we compared glomerular patterns across all tested odors per rat at the same concentration v.p. The number of correlations was similar for each concentration: *n* = 25 (0.3%), 28 (1%), 28 (3%), and 28 (10%). **Figure 5B** parametrically (t_90_) explored the similarity of the dynamics across concentrations: to what extent do the fastest versus slowest glomeruli at 1% v.p. retain their dynamic differences at the other concentrations? For each odor and rat we grouped the slowest and fastest 25% of the glomeruli (typically seven glomeruli per group) at 1% v.p. We next calculated the t_90_ of these two glomerular groups at all odor concentrations, and the difference in t_90_ between them. Next the average ± SEM t_90_ was calculated across all odors and animals per concentration (*n* = 16–17), per group, and this was reported in **Figure 5B** and used for *t*-tests. We explored the across-glomerular pattern similarities of response magnitude and dynamics between ortho- and retronasally presented odors at 4% v.p. in **Figure 5D**, based on a previously published dataset (Gautam and Verhagen, 2012b; *n* = 9 rats). Correlations were calculated across glomeruli between trial-averaged response variables per odor per animal [5–9 odors (including air) and 6–29 glomeruli per animal (150 in total), 70 correlations in total]. These correlations were used in a *t*-test (paired, 1-sided), to test if they were larger than 0. We then calculated the average correlation per animal and report their mean ± SEM across rats. *t*-tests (paired, 1-sided) were performed across rats (*n* = 9 and *n* = 4, respectively) to establish whether correlations were higher between routes than between 0.5 and log spaced concentrations.

## RESULTS

### RETRONASAL ODOR INTENSITY

We first sought to determine the effect of retronasal odor concentration on glomerular responses of the OB. By monitoring multiple ROIs across the dorsal OB, we were able to record the glomerular input activity patterns in the glomerular layer during retronasal odor presentation. **Figure 1A** shows a schematic of the setup and **Figure 1B** the resting light intensity (RLI) anatomical map of the OB. **Figures 1C1–F1** are examples of OB response maps for one rat presented with retronasal EB at 0.3, 1, 3, and 10% VP. Temporal dF/F traces are provided below each map (**Figures 1C2–F2**) for indicated ROIs (**Figure 1B**). Both the response amplitudes and the number of activated glomeruli increased with concentration, and responses occurred sooner in this example. These issues are statistically scrutinized across all concentrations, odors and rats below.

Significant effects of odor and concentration, and of their interactions, were seen across all animals when subjected to a multivariate analysis (**Table 1B**). More specifically, response amplitudes were compared across four concentrations (0.3, 1, 3, and 10% s.v.) for five odors (EB; MV; Hexa; 2-BUT; and AA) presented retronasally. Thus, response amplitudes of activated glomeruli increased as a result of odor and concentration (**Table 1B**). Please note that as rat 2 and 5 were tested with only one odor, that the statistical effect of odor is due to a reliable difference in responses between this odor and clean air, which was always tested.

Significant increases in averaged population glomerular response magnitudes were observed as the concentrations of the five odorants were systematically increased (**Figure 2A**, **Table 1**, *n* = 9 rats). Polar compounds EB (*P* = 1.85) and hexa (*P* = 1.80) displayed similar slopes (0.55 and 0.51, respectively). Conversely, AA (*P* = 2.26) and lowest polar 2-BUT (*P* = 0.26) displayed less steep slopes of 0.33 and 0.28, respectively. Odor MV (*P* = 1.85) displayed weak response at low concentrations. No consistent relationship in response magnitude was observed across odorants of different vapor pressures, polarity or molecular weight (nor was the study optimized to test for this). In summary, all retronasal odorants displayed clear significant increases in response strength, as measured by calcium-sensitive dye, with increases in odor concentration.

To evaluate the individual glomerular concentration-response functions we performed cluster analyses per odor on the trial-averaged response magnitudes (Figures S1–S5). Glomeruli with the most similar across-concentration profiles (i.e., with highest correlation) were clustered first, and their average pattern substituted the original patterns. The next most similar pattern was clustered accordingly, until all glomeruli were clustered. Glomerular clusters were next divided based on their concentration-response magnitude patterns, being either roughly linearly increasing (with maximum responses at 10% v.p.) or tuned (i.e., with a maximum response at 3% v.p. or lower). Red horizontal lines in Figures S1–S5 indicate how the glomeruli were grouped. Figure S6 provides an overview of all responses across all odors. For odor AA no glomeruli were tuned (83 glomeruli), for 2-BUT 29% of 56 glomeruli were tuned, for EB 15% of 148 glomeruli were tuned, for hexa 36% of 140 glomeruli were tuned and for MV 32% of 110 glomeruli were tuned. **Figure 2B** shows the response magnitude (mean ± SEM) of the linearly increasing and tuned glomerular groups per odor. Overall 23% of the 538 response profiles were tuned and 77% were linearly increasing with increasing concentration. Tuned glomeruli showed clear peak response magnitudes at 3% for EB and MV. For Hexa and But tuned glomeruli had roughly a flat concentration-response profile. Linearly increasing glomeruli had maximum responses at 10% v.p. (**Figure 2B**) with steeper functions than the population-averages of **Figure 2A**.

### RETRONASAL ODOR CONCENTRATION AND GLOMERULUS RECRUITMENT

To analyze recruitment of glomeruli, the threshold for activated glomeruli was determined in two different ways: clean air and noise. In the first analysis, only glomerular responses 1.65 SD above the average clean air response (0.7 ± 0.2 ΔF/F) were considered to be active. Both the normalized fraction of activated glomeruli (**Figure 3A**) and total number of glomeruli activated (**Figure 3B**) significantly increased with increasing odor concentration (**Figures 3A,B**, P < 0.01, *F*_3,16_ = 5.57, *n* = 9 rats). In the second analysis, the average pre-stimulus noise for the calcium signal (0.6 ± 0.3 ΔF/F) was calculated and responses 1.65 SD greater than the average noise level were included in the analysis (**Figure 3C**). Using this criterion, similar patterns of concentration-dependent recruitment of glomeruli were observed (*P* < 0.005, *F*_3,16_ = 6.49, *n* = 9). Thus, regardless of glomerular activation criteria, with increasing odor concentrations all retronasal odors recruited glomeruli. However, in contrast to the near monotonic increases in dF/F over the entire concentration range (**Figure 2**), recruitment saturated or peaked at 3% s.v. for some odors, 10% for the others (**Figure 3**). Increases in odor concentration can result in an increase in the number of activated glomeruli.

### TEMPORAL POPULATION DYNAMICS OF RETRONASAL ODORANTS

We evaluated effects of concentration on glomerular population temporal response profiles. The onset of glomerular calcium signals was influenced by concentration for some retronasal odorants, but not others. The relatively polar odor AA displayed a decrease in glomerular response onset times with increases in odor concentration (**Figure 4A**). **Table 2** shows that this was the only temporal parameter consistently affected by concentration for AA, despite apparently large shifts on average. EB showed significant shifts with concentration for t_10_ and t_50_, which however, were not consistent between them (**Figure 4B**). MV, also a relatively polar molecule, also reflects this trend of earlier glomerular response onset with increases in odor concentration (with exception of 0.3% s.v.; **Figure 4C**). Interestingly, 2-BUT displayed a comparatively fast response rise onset for all odor concentrations except at a 3% concentration (**Figure 4D**). Hexa did not show reliable effects of concentration on temporal parameters (**Table 2**, **Figure 4E**). Taken together, increasing concentrations do not consistently yield earlier responses.

Using strictly temporal parameters, the ability to distinguish odor concentration was possible for all odorants except Hexa. Most consistently affected were t_10_ and t_50_ (being the time to 10%, respectively 50% of peak amplitude), namely of EB, MV, and 2-BUT (*P* < 0.01, **Table 2**). On the other hand, time-to-peak did not reliable vary as a function of concentration for any odor. Temporal dynamic response patterns appear to contain information about odor concentration, although this neural coding appears to be dependent on odor. We did not find consistent correlations between vapor pressure, molecular weight or polarity and these temporal parameters (possibly due to the narrow range of these physicochemical properties of our stimulus set, which was not selected to investigate such relationships but selected to maximize dorsal OB response magnitudes).

### GLOMERULAR MAGNITUDE AND DYNAMIC PATTERNS VARY AS A FUNCTION OF ODOR CONCENTRATION

Analyses thus far focused on trends of the glomerular population as-a-whole by averaging across glomeruli for each rat. But this approach doesnot address how the patterns across glomeruli vary. We investigated this by using correlation analysis, i.e., comparing the pattern of response magnitudes and dynamics across glomeruli for each of the four rats tested with at least four odors and yielding at least 20 glomeruli (boldfaced in **Table 1A**).

The response magnitude-based patterns were quite similar between 0.5 log % v.p. differences in concentration (aggregate of 10 versus 3%, 3 versus 1%, and 1 versus 0.3%) for the same odor (**Figure 5A**, *r* = 0.57 ± 0.09), but this decreased to *r* = 0.34 ± 0.11 at a 1.5 log difference, yet all remained significantly above *r* = 0. In contrast, temporal glomerular patterns were poorly preserved across concentrations, remaining significantly correlated within only 0.5 log units and only at around *r* = 0.2. A random control (“rand”), derived in the same way as the other parameters by substituting each glomerular measurement for each rat odor and concentration with a random number is included as a reference and control against spurious correlations. These data suggest that glomeruli that respond relatively early will only to a small degree also respond earlier at other concentrations. We explored to what extent this held parametrically for t_90_ (*r* = 0.21 ± 0.08). For each odor and rat separately we determined the t_90_ for the slowest and fastest quartile of glomeruli at 1% v.p., yielding a difference of 296 ms (**Figure 5B**). This difference was reduced to a mere 96 ms at 0.3% for the same quartiles (yet significantly above 0) and 54 ms at 3% v.p. and 37 ms at 10% v.p. (ns, **Figure 5B**). This confirms that there is only a moderate yet significant conservation of the order in which glomeruli respond across concentrations.

We next explored how patterns were conserved across odors at the same concentration. Thus, whereas in **Figure 5A** we compared patterns across concentrations (expressed as log-unit differences) for the same odor, here we compared patterns across odors at the same concentration (indicated at % v.p.). Magnitude based maps (dF/F) were moderately similar at *r* ≈ 0.3–0.4 (**Figure 5C**). The temporal across-glomerular patterns were lowly correlated between all tested odors, reaching *r* = 0.2 only for t_90_ at 0.3% v.p. (*r* = 0.20 ± 0.07). Although a few reached significance, their correlations were low. At 10% v.p. temporal patterns were not correlated across odors. Thus, temporal patterns across glomeruli are poorly conserved across odors at the same concentration.

Last, we evaluated how conserved these patterns across glomeruli are between ortho- and retronasal stimulation (**Figure 5D**). We performed a new correlation analysis on a dataset used for a prior publication (Gautam and Verhagen, 2012b) based on 150 glomeruli from 9 rats, each presented with 4–8 odors at 4% v.p. multiple times each route. Response maps (dF/F) were fairly similar between the routes (*r* = 0.53 ± 0.10). Interestingly, especially late dynamics were fairly well conserved, with e.g., t_peak_ correlated at *r* = 0.42 ± 0.05. Additionally, late temporal patterns were significantly more similar between odor routes than between retronasal odors of 0.5 log concentration difference (**Figure 5D**), even though the retronasal response magnitude was only 63% of the orthonasal magnitude (Gautam and Verhagen, 2012b).

## DISCUSSION

Glomerular activity patterns contain information about odor properties. Only recently was it found that rodents display unique patterns of glomerular activity, in response to the same odor presented orthonasally versus retronasally (Gautam and Verhagen, 2012b). Work presented here is the first to systematically characterize concentration response curves of calcium activity in the glomerular layer in response to retronasal odor stimulation. Glomerular activity patterns were monitored *in vivo* in response to retronasal odor stimulation to identify stimulus specific calcium responses that may underlie fundamental aspects of olfactory network processing.

Data presented here show a significant positive relationship between odor concentration and response magnitude across all tested retronasal odorants. Previous work that examined several odorants, including 2-hexanone and 2-BUT, also found orthonasal response magnitudes to increase with odor concentration and the reported Hill coefficients ranged from 0.5 to 1.9 (Wachowiak and Cohen, 2001). Other work found that responses to increasing concentrations of retronasal valeric acid (dilutions in mineral oil which need not scale linearly with flow dilutions) in 1 mouse increased in 5 of 9 glomeruli that all responded when presented orthonasally (Furudono et al., 2013). Our data show that linearly fitted (*r*^2^ > 0.85) retronasal logarithmic concentration-response curves yields slopes that range from 0.28 to 0.55 (**Figure 2A**). Thus for a 10-fold odor concentration increase we predict between a 0.28% dF/F (2-But) to up to a 0.55% dF/F increase in response magnitude.

Although increases in odor concentration resulted in corresponding increases in averaged glomerular response magnitudes and glomeruli recruitment across all odors (**Figures 2 and 3**), the concentration-response magnitude functions differed between odors (**Figure 2**). We also found evidence of tuned response profiles (23% overall), but this too varied (between 0 and 36%) across odors (**Figure 2B**, Figures S1–S6). Thus, the input of the dorsal OB shows odor-dependent concentration-response functions, both at the glomerular and population level.

We previously reported that retronasal population-averaged responses were generally slower than their orthonasal counter parts and we also showed less temporal variability across retronasal odors than orthonasal odors 4% v.p. (Gautam and Verhagen, 2012b). Here we report significant effects of concentration on temporal population-averaged profiles for all but one odor (**Figure 4**, **Table 2**). Thus, for retronasal odors the effect of concentration on population-averaged dynamics is stronger than that of odor quality, though in a non-uniform way. It is in this light not surprising that there was little similarity among glomerular temporal patterns across concentrations (**Figures 5A,B**) and even less so across retronasal odors (**Figure 5C**). Indeed, the dynamics were more similar between routes at the same concentration than across concentrations differing only threefold for the same retronasal odor (**Figure 5D**).

Thus, with varying concentration different retronasal odors show different individual and population glomerular response magnitude modulation and this occurs in combination with different glomerular dynamics. The mechanisms are unknown to our knowledge, but may involve differences in odor polarity and vapor pressure and non-uniform receptor distributions (Schoenfeld and Cleland, 2006). This has interesting implications for complex odor mixtures found in food, the concentrations of which vary greatly and vary across time. We hypothesize that both the decorrelated concentration-dynamics functions -as suggested before (Stopfer et al., 1997)- and odor-dependent concentration-response functions can help the bulbar circuit to decorrelate the odor input. The temporal dynamics of odor qualities and concentrations may be disambiguated in downstream olfactory network neurons (Haddad et al., 2013).

We find it hard to reconcile the fact that retronasal odors evoke 50–60% of the response magnitude of orthonasal odors (Gautam and Verhagen, 2012b), being equivalent to a ∼10× concentration difference (**Figure 2**), and that there was no significant temporal correlation across glomeruli at such a 10-fold difference (**Figure 5A**), with the finding that temporal patterns can be quite similar between the ortho- and retronasal route (**Figure 5D**). The data from this and our prior publication were not based on the same rats, however, the rats were of the same strain and sex and the experimental setup and experimentalists were also the same. Assuming noise is not the cause, as we do find strong population level effects of concentration on magnitude and dynamics (**Tables 1** and **2**) and also among magnitude correlations (**Figure 5A**), we pose that this apparent dichotomy is a real biological phenomenon. We propose that the difference in odor route somehow compensates for the associated difference in efficacy to conserve temporal fidelity across glomeruli for a given odor concentration.

Our previous work examining differences in glomerular responses to retronasal and orthonasal stimulation found that less volatile odorants create relatively smaller retronasal glomerular responses. The volatility of the odor molecule did not appear to influence the temporal response patterns of glomerular activity across both retronasal and orthonasal stimulation (Gautam and Verhagen, 2012b). Recent findings show strong positive correlations with the molecular weight of odors and the magnitude of glomerular as well as mitral cell responses in orthonasal responses of rats (Wojcik and Sirotin, 2013). The large stimulus set we employed here, however, precluded us from presenting the same stimuli both retro- and orthonasally. Further, the odorants were chosen for maximum OB response magnitude, not to evaluate physico-chemical correlates.

It could be argued that, while this is the first study to specifically and systematically look at retronasal responses, it is not the first to have included retronasal responses. Prior studies that used free breathing animals while presenting orthonasal odorants at the nares may also have included contributions from retronasal responses upon subsequent odor exhalation. This does not pertain to studies where an orthonasal-direction-only vacuum (“artificial sniffing”) is used. Of those free breathing studies, the odor source was at the nares, not the mouth or pharynx (as in this study). This implies that after passing through the nose and the lungs, only a fraction (42 ± 15%, mean ± SD across 38 volatile organic compounds) is returned to enter the nasal cavity retronasally due to lung retention (calculations based on Tables 1 and 2 in Jakubowski and Czerczak, 2009). Further, we previously showed that retronasal dorsal OB responses are ∼50–60% of orthonasal responses, when stimuli are presented at the nasopharynx and nares, respectively, and lung retention is thereby avoided (Gautam and Verhagen, 2012b). Hence, the OB responses in the free breathing studies mentioned are dominated by orthonasal responses due to the compounding effects of lung retention and low relative retronasal sensitivity and further do not provide route-specific information.

Work shown here definitively shows that retronasal odor response patterns in the dorsal OB, like orthonasal odor response patterns, depend not only on odor (quality code), but also its concentration (intensity code). Increases in retronasal odor concentration increase the response magnitude of the activated glomeruli, and recruit additional glomeruli. Retronasal odor concentration also influences the temporal dynamics of glomerular responses. Interestingly, all of these effects differ across odors, indicating that a complex glomerular code is involved in coding food flavor.

## Conflict of Interest Statement

The authors declare that the research was conducted in the absence of any commercial or financial relationships that could be construed as a potential conflict of interest.

## SUPPLEMENTARY MATERIAL

Figure S1 **Concentration-response profiles and cluster analysis.** Heatmap of individual glomerular responses across odor concentrations organized vertically by hierarchically clustered across-glomerular concentration-response profile similarities (averaged Pearson correlations). Dendrogram shown on the right. Legend indicated response magnitudes. White cells have no data. Horizontal red line(s) separate clusters with different concentration-response profiles (linear: maximum response at 10% v.p., tuned: maximum response below 10% v.p.). **Figure 2B** shows the mean response magnitude for the identified clusters.

Figure S2 **Concentration-response profiles and cluster analysis.** Same as **Figures S1** but for butanone.

Figure S3 **Concentration-response profiles and cluster analysis.** Same as **Figures S1** but for ethyl butyrate.

Figure S4 **Concentration-response profiles and cluster analysis.** Same as **Figures S1** but for hexanal.

Figure S5 **Concentration-response profiles and cluster analysis.** Same as **Figures S1** but for methyl valerate.

Figure S6 **Concentration-response profiles and cluster analysis.** Similar to **Figures S1** but for all odors. Clustering in this case was based on Euclidian distances. Response profiles across odor concentrations were also clustered (bottom dendrogram).
