# Supplementary figures and images for: Retronasal odor concentration coding in glomeruli of the rat olfactory bulb
Source: Front Integr Neurosci. 2014 Oct 24;8:81. doi: 10.3389/fnint.2014.00081 (PMC4208450; doi:10.3389/fnint.2014.00081)

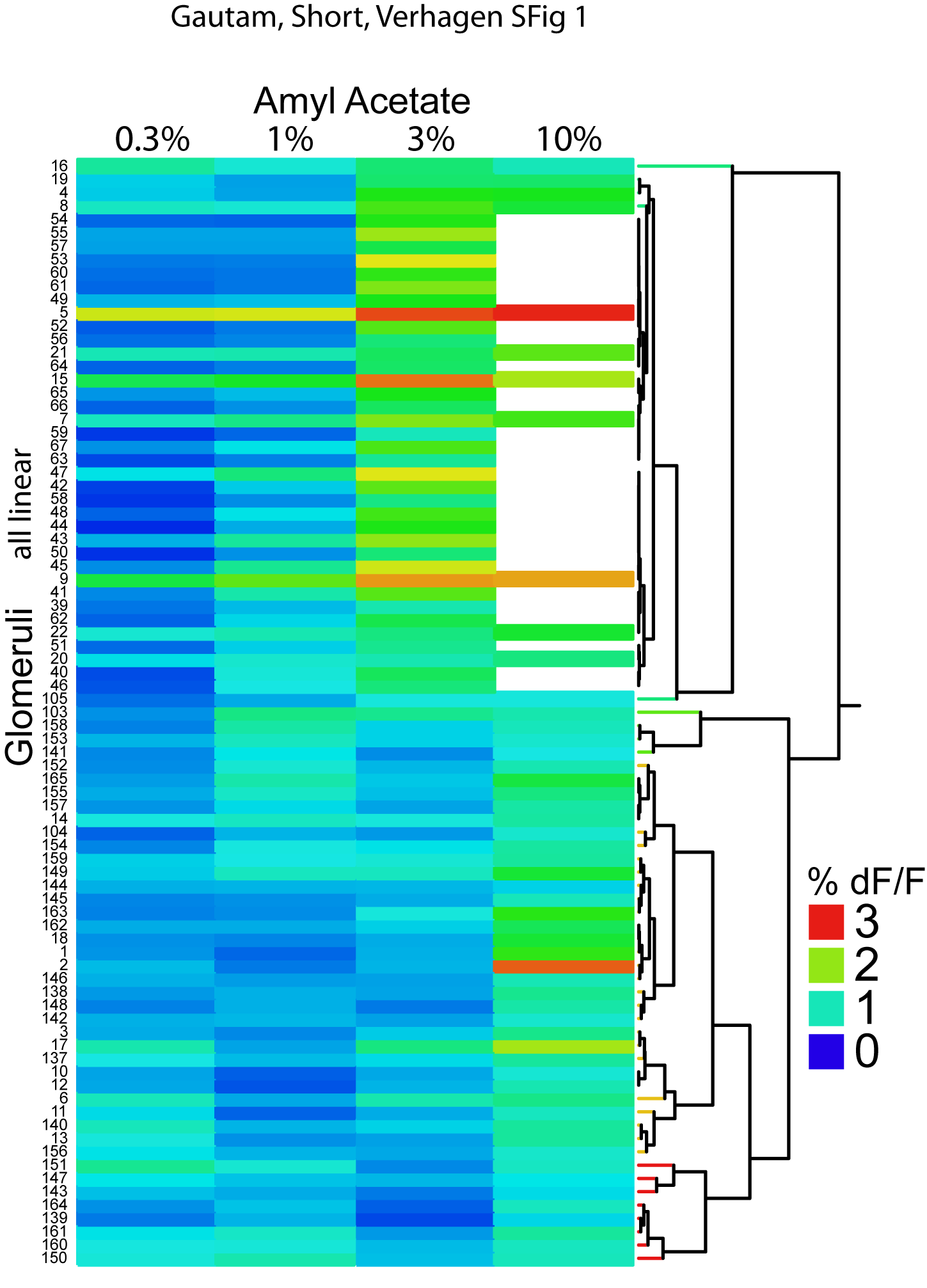

Supplement: Figure S1 — Concentration-response profiles and cluster analysis. Heatmap of individual glomerular responses across odor concentrations organized vertically by hierarchically clustered across-glomerular concentration-response profile similarities (averaged Pearson correlations). Dendrogram shown on the right. Legend indicated response magnitudes. White cells have no data. Horizontal red line(s) separate clusters with different concentration-response profiles (linear: maximum response at 10% v.p., tuned: maximum response below 10% v.p.). Figure 2B shows the mean response magnitude for the identified clusters. [file Image_1.TIF]

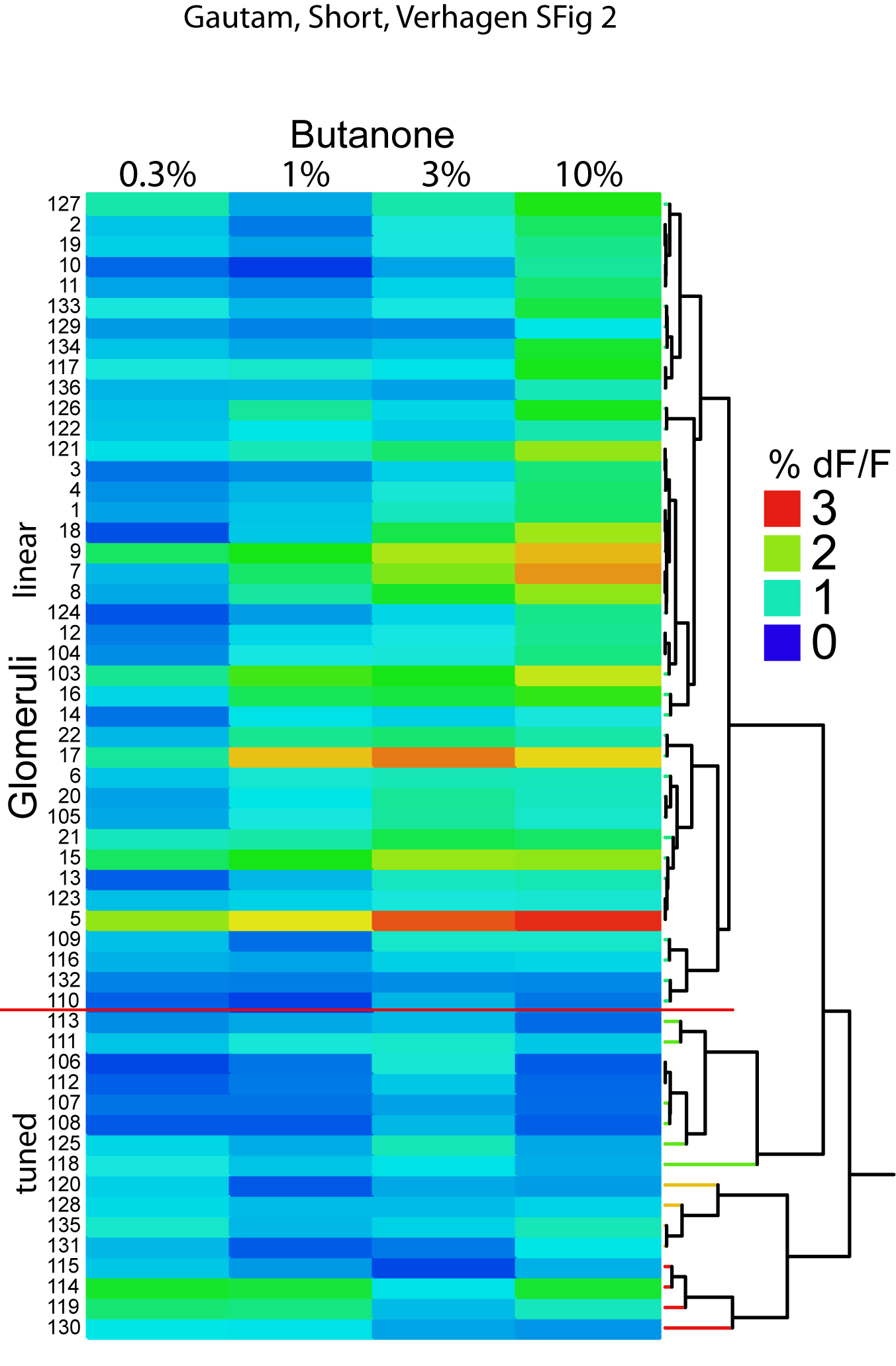

Supplement: Figure S2 — Concentration-response profiles and cluster analysis. Same as Figures S1 but for butanone. [file Image_2.TIF]

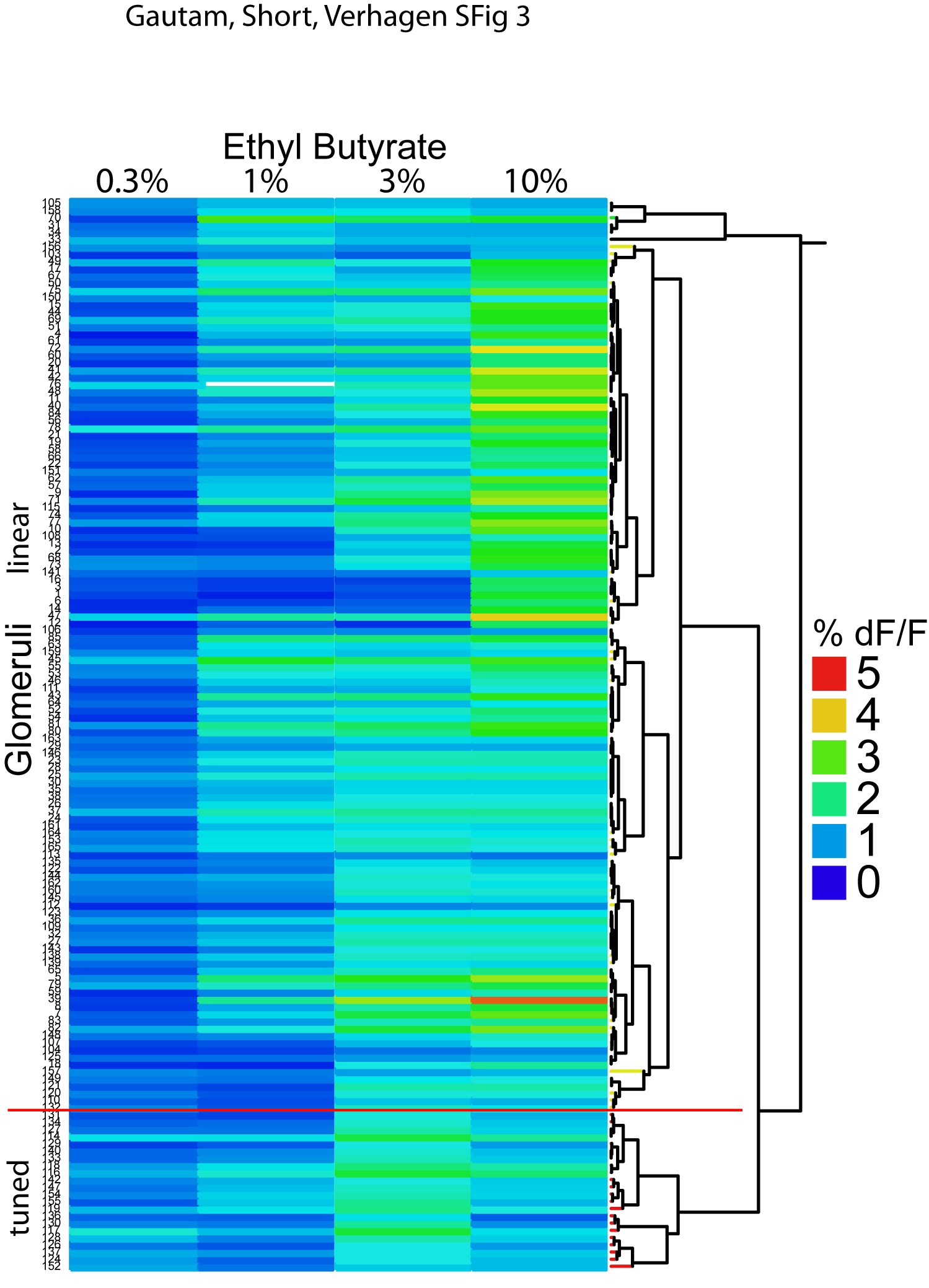

Supplement: Figure S3 — Concentration-response profiles and cluster analysis. Same as Figures S1 but for ethyl butyrate. [file Image_3.TIF]

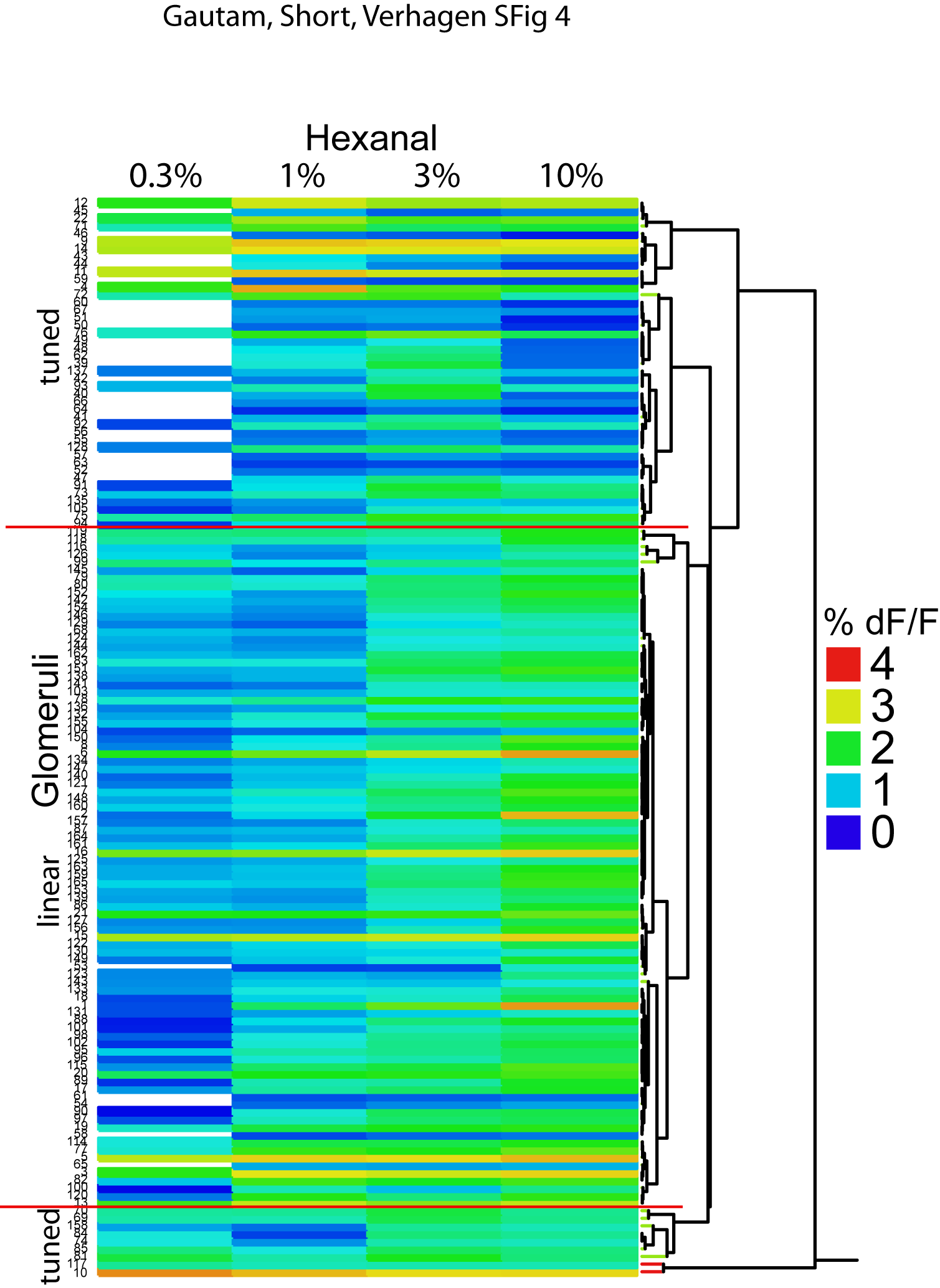

Supplement: Figure S4 — Concentration-response profiles and cluster analysis. Same as Figures S1 but for hexanal. [file Image_4.TIF]

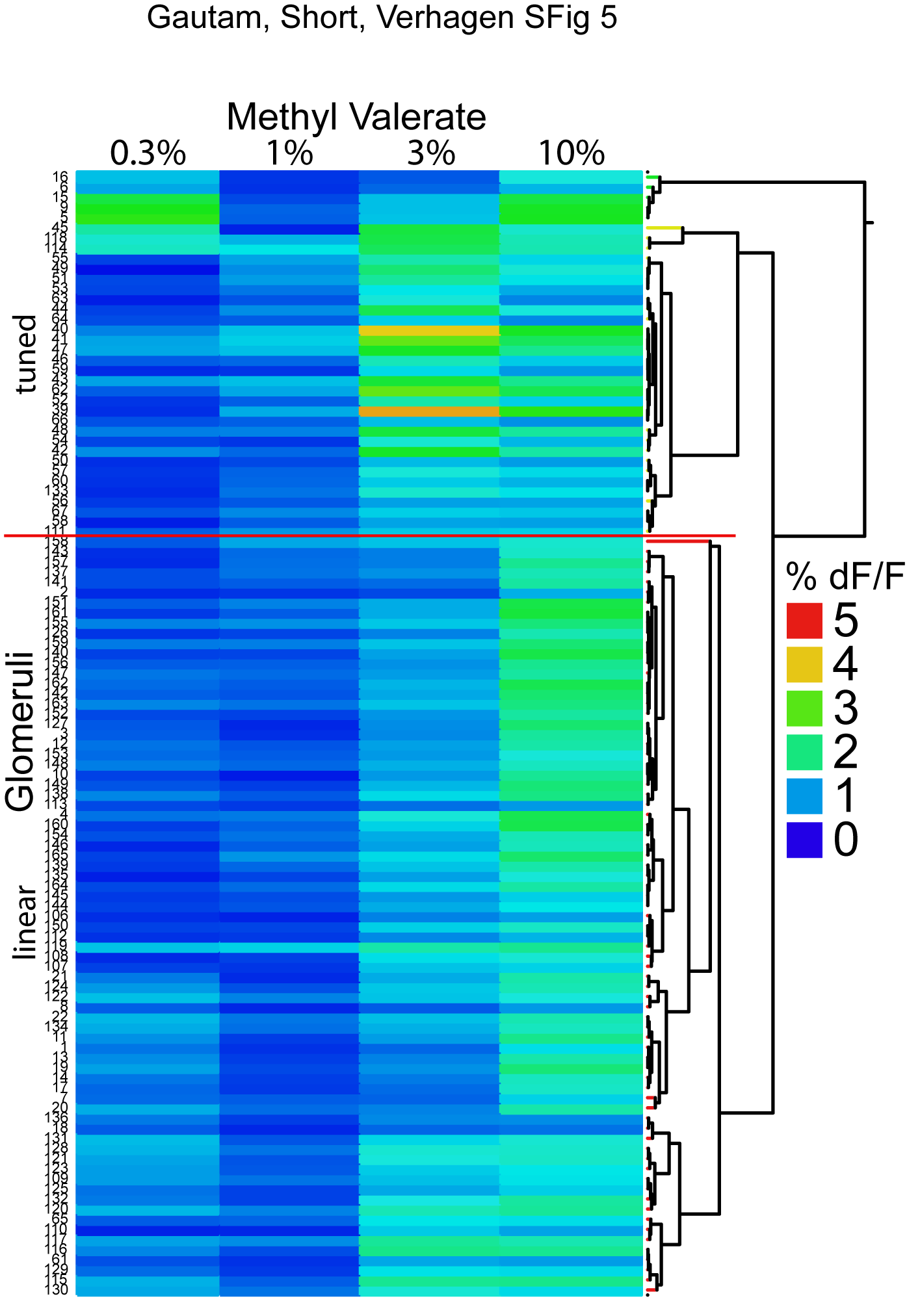

Supplement: Figure S5 — Concentration-response profiles and cluster analysis. Same as Figures S1 but for methyl valerate. [file Image_5.TIF]

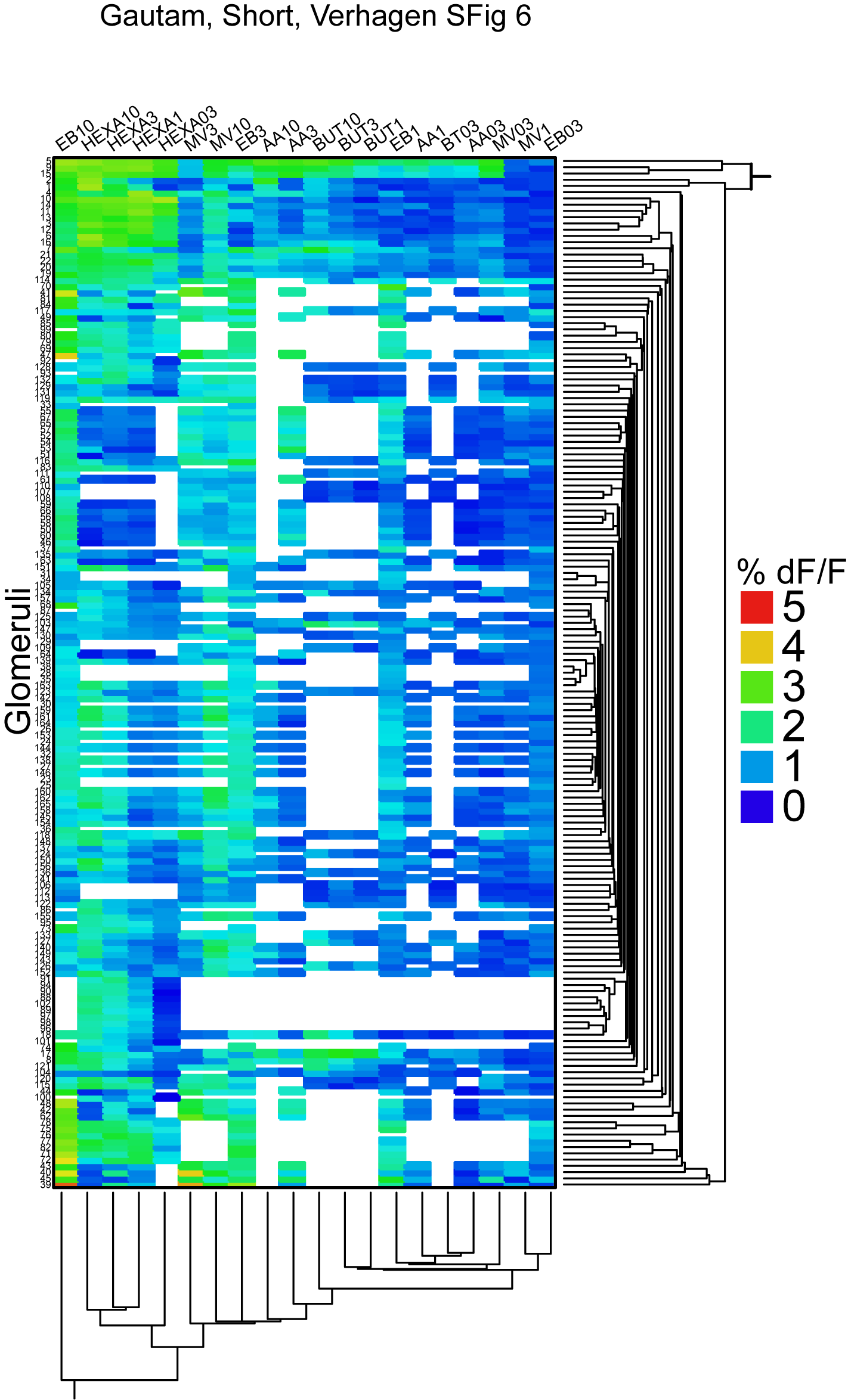

Supplement: Figure S6 — Concentration-response profiles and cluster analysis. Similar to Figures S1 but for all odors. Clustering in this case was based on Euclidian distances. Response profiles across odor concentrations were also clustered (bottom dendrogram). [file Image_6.TIF]
